# Characterization and Source Investigation of Multidrug-Resistant *Salmonella* Anatum from a Sustained Outbreak, Taiwan

**DOI:** 10.3201/eid2612.200147

**Published:** 2020-12

**Authors:** Ye Feng, Yi-Jung Chang, Shih-Chuan Pan, Lin-Hui Su, Hsin-Chieh Li, Hsin-Ping Yang, Min-Jia Yu, Cheng-Hsun Chiu

**Affiliations:** Sir Run Run Shaw Hospital, Zhejiang University School of Medicine, Hangzhou, China (Y. Feng);; Institute for Translational Medicine, Zhejiang University School of Medicine, Hangzhou (Y. Feng);; Key Laboratory of Microbial Technology and Bioinformatics of Zhejiang Province, Hangzhou (Y. Feng);; Division of Pediatric Infectious Diseases, Chang Gung Memorial Hospital, Chang Gung University College of Medicine, Taoyuan (Y.-J. Chang, S.-C. Pan, C.-H. Chiu);; Molecular Infectious Disease Research Center, Chang Gung Memorial Hospital, Chang Gung University College of Medicine, Taoyuan, Taiwan (Y.-J. Chang, L.-H. Su, H.-C. Li, H.-P. Yang, M.-J. Yu, C.-H. Chiu)

**Keywords:** *Salmonella* Anatum, multidrug resistance, outbreak, retail meats, foodborne illness, bacteria, Taiwan, food safety

## Abstract

An ongoing outbreak of multidrug-resistant *Salmonella enterica* serovar Anatum began in Taiwan in 2015. Pork and poultry were identified as vehicles for transmission. Contaminated meat contributed to the high rate of infections among children. Nearly identical *Salmonella* Anatum strains have been identified in the United Kingdom, the United States, and the Philippines.

Nontyphoidal *Salmonella* (NTS) is a major cause for foodborne diseases worldwide. In Taiwan, the ambient climate and flourishing pig-raising industry makes NTS infections rampant. As in other countries, salmonellosis was primarily caused by *Salmonella enterica* serovars Enteritidis and Typhimurium in Taiwan (*1*), but rare serovars such as *Salmonella* Goldcoast have appeared in recent years (*2*). Recommended antimicrobial treatment options for salmonellosis include fluoroquinolones and extended-spectrum cephalosporins (*1*). However, resistance to these antibiotics has been emerging in many countries, leading to increased disease prevalence, disease severity, and death and the requirement of last-line antimicrobial drugs (e.g., carbapenems) (*3*–*5*).

Since 2015, northern Taiwan has seen an increase in *Salmonella* infections, caused by previously rare *Salmonella* Anatum. The infections were also reported in central Taiwan, indicating that this outbreak had already prevailed throughout the entire island (*6*). Co-resistance to ceftriaxone and ciprofloxacin are the main feature of the outbreak clone. Evidence from epidemiologic, laboratory, and supply-chain investigations identified raw pork and poultry as the vehicle for spread of this strain. More important, genomic comparisons against the global public database indicated that this clone has appeared in Europe, Asia, and America. Given the increasing globalization of foodstuffs, these findings prompt an urgent global sharing of whole-genome sequencing (WGS) data to facilitate disease surveillance and early recognition of international foodborne outbreaks (*7*,*8*).

## The Study

**Figure 1:**
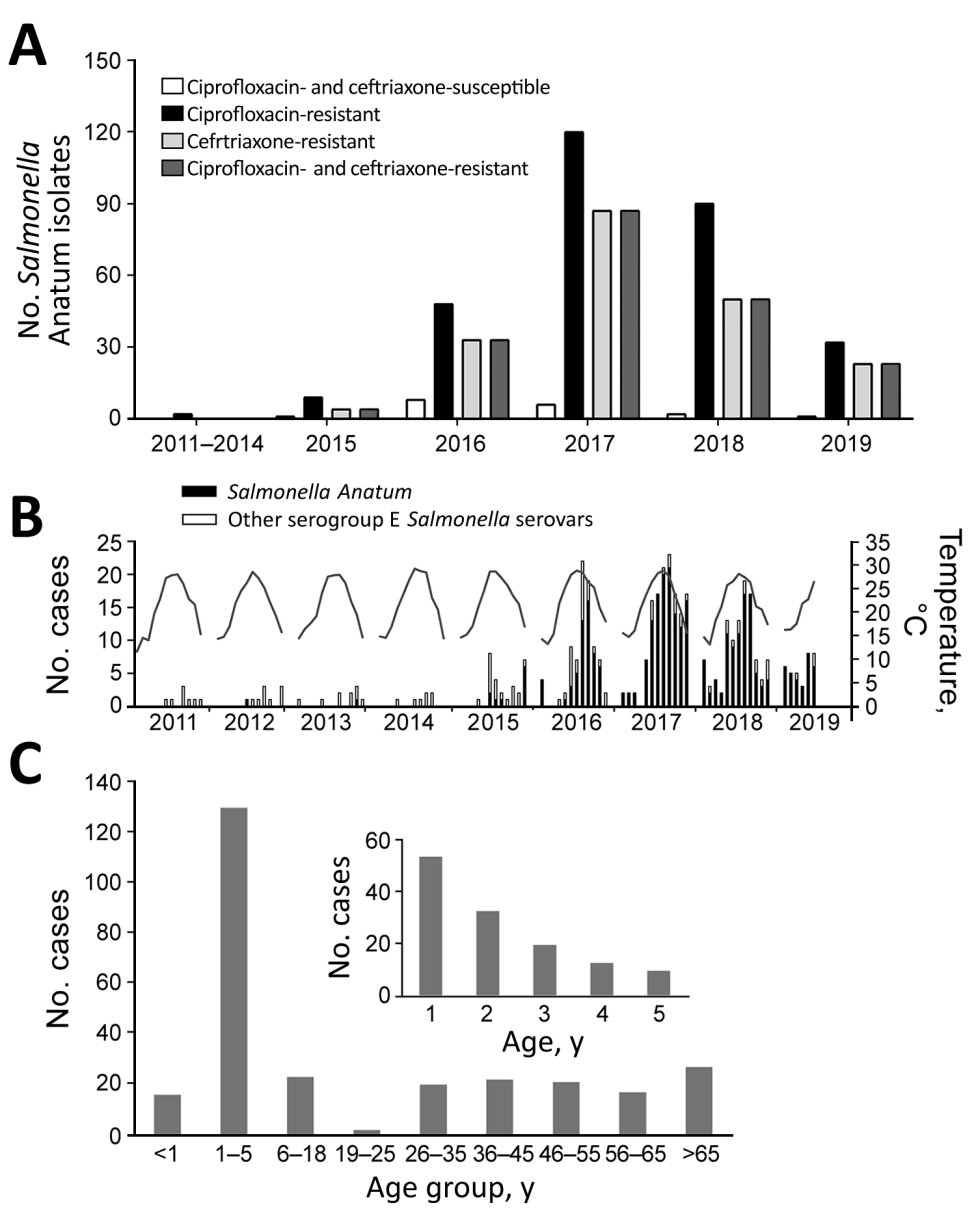
*Salmonella enterica* serotype Anatum infection and antimicrobial resistance, Taiwan. A) Antimicrobial resistance of the *Salmonella* Anatum isolates collected in Chang Gung Memorial Hospital. B) Monthly case number (bar plot) and temperature (line). C) Age distribution of patients diagnosed during 2015–2018.

Chang Gung Memorial Hospital is a main referral hospital for cities in northern Taiwan, including Taipei, New Taipei, and Taoyuan. The population in this region is »7 million. In 2012, the hospital’s clinical microbiology laboratory launched a program to monitor the NTS serovars causing human infections. All *Salmonella* isolates from patients were collected and serotyped. Before 2015, very few *Salmonella* Anatum isolates were recovered, and most were susceptible to antimicrobial agents. Since then, an increase has been observed, peaking in 2017 (Figure 1, panel A). As of June 2019, a total of 319 nonrepetitive isolates have been identified; of these, 197 (61.8%) isolates were ceftriaxone-resistant (MIC >2 μg/mL), 301 (94.4%) were ciprofloxacin-resistant (MIC >0.12 μg/mL), and 197 (61.8%) were resistant to both. In addition, 292 (91.5%) isolates were resistant to chloramphenic, and 295 (92.5%) were resistant to trimethoprim/sulfamethoxazole. A positive correlation was found between higher temperatures and the infections (*r* = 0.4; p<0.05) (Figure 1, panel B); however, no notable effects on *Salmonella* Anatum infections have been associated with precipitation or humidity (*r*<0.3; p>0.05).

Detailed methods are described in the Appendix. We first reviewed the clinical and laboratory characteristics of 278 patients from 2015–2018. Most patients had acute gastroenteritis, whereas a few (14/278, 5%) had invasive diseases, such as bacteremia and sepsis. In terms of age distribution, the highest number of cases were in young children (Figure 1, panel C). Pediatric patients (n = 169) had significantly higher rates than adult patients (n = 109) for hospitalization (79.2% vs. 55.0%; p<0.05), diarrhea (89.9% vs. 68.8%; p<0.05), and fever (89.2% vs. 58.1%; p<0.05).

**Figure 2:**
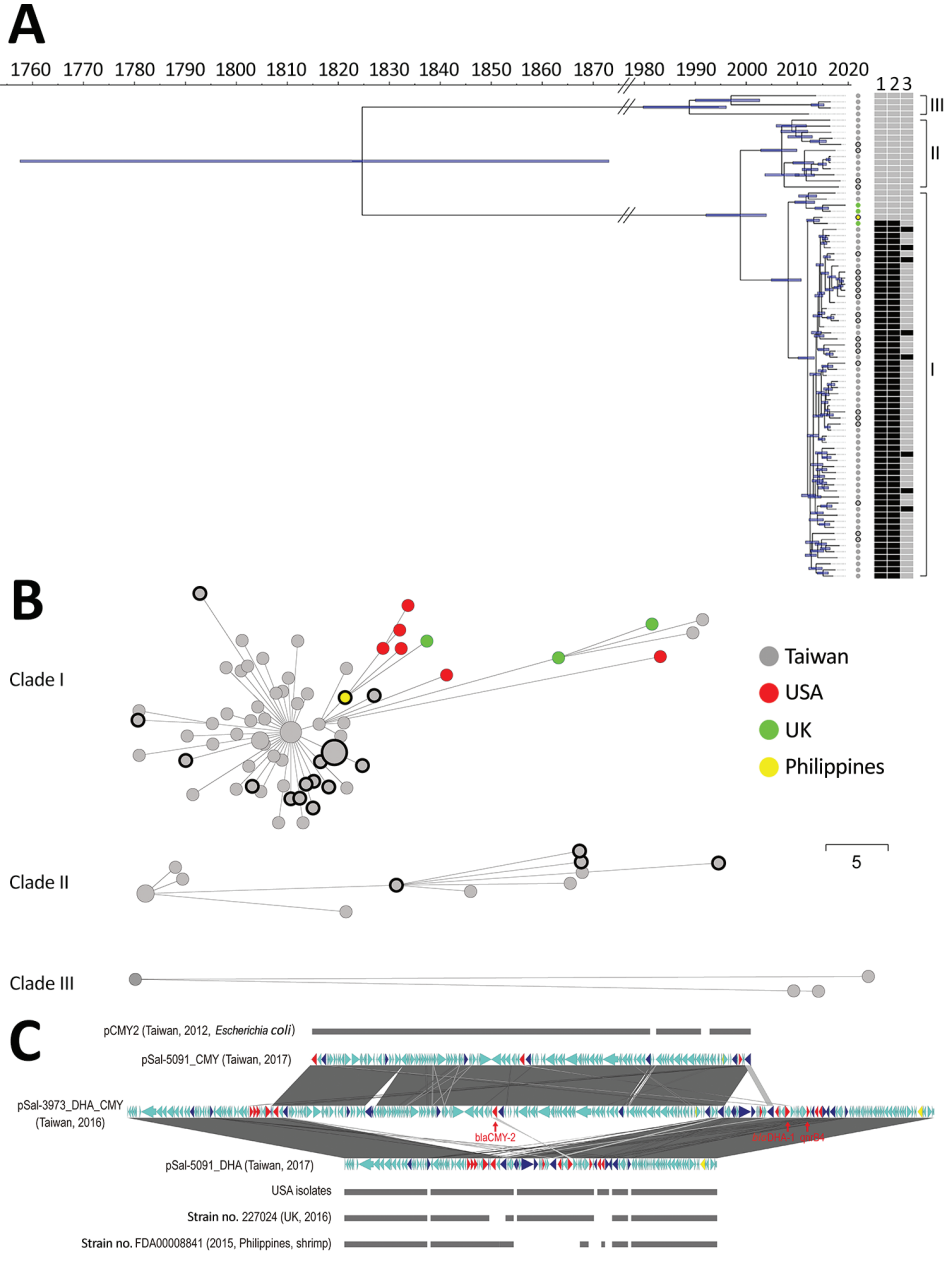
Genomic analysis of the outbreak caused by *Salmonella* *enterica* serotype Anatum, Taiwan. A) Dated phylogeny for *Salmonella* Anatum clinical isolates and food and environmental isolates. All isolates were divided into 3 clades, shown at right. The nodes’ colors represent the geo source; nodes with black rings were from meat or the environment, and the remainder were derived from the patients. The right heatmap represents the presence (in black) or absence (in gray) of key antimicrobial-resistance genes (1, *bla*_DHA-1_; 2, *qnrB4*; 3, *bla*_CMY-2_). B) Minimal spanning tree based on alleles identified through core genome multilocus sequence typing. Dots with black circles represent food isolates; the others are clinical isolates. The collection date for the 6 US isolates in panel B was missing in GenBank and therefore not included in panel A. Scale bar indicates 5 single nucleotide polymorphisms. C) Gene structure of multidrug-resistant plasmids in *Salmonella* Anatum in Taiwan compared with international isolates. Two types of plasmids were identified in the clade I *Salmonella* Anatum isolates in Taiwan. One carried *bla*_CMY-2_, with its structure being shown by pSal-5091_CMY. A similar plasmid, pCMY2 (GenBank accession no. LC019731.1), is shown. The other carried *bla*_DHA-1_; its structure is shown by pSal-5091_DHA. International isolates shown in the figure, whose genomes also were downloaded from GenBank (Appendix), possess very similar plasmids. In certain isolates, the 2 plasmids can integrate into 1 large plasmid, with its structure shown by pSal-3973_DHA_CMY. Red genes represent antimicrobial-resistance genes; blue genes represent transposase/integrase genes; and yellow genes represent Inc-determinant genes.

Multilocus sequence typing indicated that the entire collection of clinical *Salmonella* Anatum isolates belonged to sequence type 64. We randomly selected 54 clinical isolates for WGS (Appendix). Both core genome multilocus sequence typing and whole-genome single-nucleotide polymorphism analyses, performed by using the BacWGSTdb database (*9*), further divided these isolates into 3 clades (Figure 2, panel A, B). Clades I and II were more closely related to each other; their most recent common ancestor occurred >21 years ago. Clade III was more distantly connected to these 2 clades. Typing based on PCR assay was performed on the unsequenced isolates. Clade I accounted for 95.6% (305/319) of all isolates, suggesting it was the cause of the outbreak. The isolates resistant to ceftriaxone, ciprofloxacin, or both clustered within clade I, whereas the isolates of clades II and III were more susceptible. Most of the clade I isolates harbored a 90-kb IncA/C plasmid carrying *bla*DHA-1 (encoding a class C β-lactamase) and *qnrB* (conferring resistance to quinolones). A conjugation assay demonstrated that this plasmid conferred ceftriaxone and ciprofloxacin resistance. In addition, 31 (9.7%) clinical isolates carried *bla*_CMY-2_, which was located within a >100-kb IncI1 plasmid and also encoded a class C β-lactamase. These 31 isolates carried *bla*_DHA-1_ simultaneously. In 11 of them, the *bla*_DHA-1_–carrying and *bla*_CMY-2_–carrying plasmids were fused into 1 large plasmid (Figure 2, panel C).

By comparing these findings against sequences in GenBank, we found nearly identical genomic sequences for isolates in the United Kingdom, the United States, and the Philippines. The collection time for these isolates also occurred during 2015–2019, which nearly coincided with the outbreak in Taiwan. These international *Salmonella* Anatum isolates also carried the 90-kb IncA/C plasmid (Figure 2, panel A, C); therefore, they were likely ceftriaxone- and ciprofloxacin-resistant concomitantly. The only distinction of these international isolates was their lack of the *bla*_CMY-2_–carrying plasmid. Accordingly, we speculated that the *Salmonella* Anatum clone had arrived in Taiwan through food trade and later acquired the *bla*_CMY-2_–carrying plasmid.

To trace the source of *Salmonella* Anatum, we investigated food samples from supermarkets and traditional markets of 8 districts with high density of *Salmonella* patients in New Taipei City and Taoyuan City, Taiwan (Appendix). A total of 11 *Salmonella* Anatum isolates were collected from pork, 4 from poultry, and 1 from beef in these regions (Appendix). WGS showed that they all belonged to clades I and II, providing strong evidence that raw meats were the outbreak vehicle. All 16 isolates harbored the *bla*_DHA-1_–carrying IncA/C plasmid. Other *Salmonella* serovars also were detected in this investigation. The overall *Salmonella* isolation rate from retail meats was significantly higher in traditional markets than in the supermarkets (p<0.001) (Appendix). In Taiwan, pork in the supermarkets is usually provided through the cold transportation chain, whereas for traditional markets pork is usually provided through the traditional chain, with notable differences. Temperatures were much lower in the cutting factory and butcher shop in the cold chain than in the traditional chain (Appendix). Furthermore, pork was wrapped by plastic tissue and bags in the cold chain, but the traditional chain did not do any wrapping or packaging during transportation.

To clarify the contradictory findings that most infections occurred in young children even though pork is not a major food for infants, we conducted a questionnaire survey among parents of 20 infants (<1 year of age) with NTS infections and 80 parents of infants without (controls) (Appendix). Parents of the infected infants more often touched, rinsed, and cooked meat before feeding other foods to their infants (Appendix). Moreover, these parents were more willing to purchase meat from traditional markets rather than supermarkets. A possibility is that they bought meat from the traditional markets, then their frequent rinsing flushed the *Salmonella* on the surface of the meats, cutting boards and knives, and sinks, and finally onto fresh vegetables, fruit, and other ready-to-eat foods that were cross-contaminated and reached the infants through parents or other caregivers. This transmission mode is of particular importance in infants and has already been reported for other bacterial pathogens such as *Yersinia enterocolitica* (*10*).

## Conclusions

Our study sought to describe an outbreak in Taiwan caused by a multidrug-resistant *Salmonella* Anatum clone. The questionnaire and supply-chain investigations we conducted found that the infection cases were closely associated with improper packaging during transportation and unhygienic food handling in the customers’ kitchen. The high similarity of genomic sequence between the Taiwan isolates and international isolates indicates the global dissemination of this clone and highlights the public health value of multicountry sharing of epidemiologic, trace-back, microbiologic, genomic, and food trade data.

Appendix Characterization and source investigation of multidrug-resistant *Salmonella* Anatum from a sustained outbreak, Taiwan.
