# Supplementary material for: Characterization and Source Investigation of Multidrug-Resistant Salmonella Anatum from a Sustained Outbreak, Taiwan
Source: Emerg Infect Dis. 2020 Dec;26(12):2951–5. doi: 10.3201/eid2612.200147 (PMC7706963; doi:10.3201/eid2612.200147)
Supplement: Appendix — Characterization and source investigation of multidrug-resistant Salmonella Anatum from a sustained outbreak, Taiwan. [file 20-0147-Techapp-s1.pdf]

Article DOI: https://doi.org/10.3201/eid2612.200147

Characterization and Source Investigation of Multidrug-Resistant *Salmonella* Anatum from a Sustained Outbreak, Taiwan

Appendix

Supplementary Methods

Patients and Setting

Chang Gung Memorial Hospital (CGMH) is a main referral hospital for cities in northern Taiwan, including Taipei, New Taipei, and Taoyuan. The population in this region is approximately seven million. The Clinical Microbiology Laboratory has launched a program to monitor the serovars of NTS causing human infections since 2012. All *Salmonella* isolates from patients were collected and serotyped. Antimicrobial susceptibility testing was performed using the disc diffusion method specified in the Clinical and Laboratory Standards Institute (CLSI) guidelines (*1*). *S.* Anatum is a relatively uncommonly recorded serovar for human infections. Prior to 2015, very few *S.* Anatum isolate was recovered and most of the isolates were susceptible to antimicrobial agents. However, since 2015, culture-confirmed infections caused by *S.* Anatum has been increasing (Figure 1, https://wwwnc.cdc.gov/EID/article/26/12/20-0147-F1.htm). A review on the clinical manifestations of the patients with *S.* Anatum infection from 2015 to 2018 was carried out. This study was approved by the Institutional Review Board of CGMH (201601804B0 and 201702155B0).

Serogrouping and serotyping was conducted as described previously (*2*). The MIC of CIP and CRO on these isolates was determined by E-test and interpreted according to the recommendations provided by CLSI (*1*).

To trace the source of *S.* Anatum, we investigated food samples from supermarkets and traditional markets of eight districts with high density of *Salmonella* patients in New Taipei City, and Taoyuan City in Taiwan (Appendix Figure 1). All the *Salmonella* isolates derived from food

samples were further examined for their serogroups, serovars, and their antimicrobial susceptibility to CRO and CIP.

Meteorological data, including temperature, humidity and precipitation in Northern Taiwan, were collected from the Taiwan Central Weather Bureau, which is available at: https://e-service.cwb.gov.tw/HistoryDataQuery/index.jsp. The correlation and lag effect of case number and temperature were analyzed using bivariate correlation and linear regression softwares in IBM SPSS Statistics version 20.0 (IBM Corp., Armonk, NY), and significance was set at $p < 0.05$.

Meat Processing and Transport

To clarify the discrepancy of *Salmonella* detection rate between different markets, and to further investigate suspicious contamination nodes during meat processing and transportation, we designed an experiment to contrast cold chain with traditional chain transportation of the meat. Four nodes at each of the transportation lines were investigated: slaughter houses (samples obtained from the carcass surfaces), cutting factories and pre-cooling chambers, where the pig carcass were cut into large pieces (samples from the environmental and meat surfaces),transport vehicles (samples from the environment), and butcher shops, where the large pork chunks were cut into small pieces for consumer purchase (samples from the environment and meat surfaces). We collected carcass surface samples and environment samples from floor, hook, conveyor belt, meat grinder, basket, kitchen knife, chopping board and workers' hands surface in slaughterhouse, cutting factory, transport vehicle and butcher shop. Each sampling surface area of the carcass and environment samples was 100 cm^2. In addition, each of the meat samples was collected over 25 g in cutting factory and butcher shop. Meat isolation and identification followed the method of Taiwan Food and Drug Administration. Carcass surface and environment sampling followed USDA Laboratory Guidebook (https://www.fsis.usda.gov) with a sensitivity of 1.4×10^1 CFU/100 cm^2. All investigated samples were collected with sterilized sponges, bags, gloves and templates (Nasco Whirl-Pak, USA).

Whole-Genome Sequencing and Conjugation

A total of 76 isolates were subjected to whole genome sequencing (WGS), that was performed using the Illumina Miseq platform (Illumina, CA, USA) and/or MinION Sequencer (Nanopore, Oxford, UK). The genome assembly and comparative analysis followed the methods

as described previously (*2*). The genomic sequences were deposited into the GenBank database, with the accession numbers being listed in Appendix Table 1. The NCBI Pathogen Detection Service (https://www.ncbi.nlm.nih.gov/pathogens) was searched for *S.* Anatum genomes that were highly similar to the genome sequences collected in Taiwan. Multilocus sequence typing was performed on all *S.* Anatum isolates (*2*,*3*).

To investigate the transferability of the resistance plasmids identified in the genome sequencing, we carried out a conjugation assay using *E. coli* J53 (a sodium-azide-resistant strain) as the recipient and outbreak *S.* Anatum isolates as the donor (Sal-4377 and Sal-4162). The conjugation assay was conducted following the protocol described in a previous study (*2*).

Questionnaire Investigation

Caregivers of children with culture-confirmed *S.* Anatum infection were invited to complete a questionnaire. The questionnaire is designed based on a WHO protocol (https://www.who.int/immunization/diseases/rotavirus/generic_protocols/en/). The total number of the questionnaire completed was 100, and the ratio of the patient to healthy controls was 1:4, namely 20 children in the patient group and 80 in the healthy group. The healthy children were recruited from well-baby clinics. Ages (<1y) were matched between the case and control groups. Chi-square test was used to analyze all questionnaire data using IBM SPSS Statistics version 20.0 (IBM Corp., Armonk, NY). The false-discovery rate (FDR) correction was made for multiple comparisons, with 0.1 as the significance threshold.

References

1. Clinical and Laboratory Standards Institute. Performance standards for antimicrobial susceptibility testing: twenty-first informational supplement. CLSI document M100–S28. Wayne (PA): The Institute; 2018.

2. Feng Y, Chang YJ, Fang SH, Su LH, Li HC, Yang HP, et al. Emergence and evolution of high-level cephalosporin-resistant *Salmonella* Goldcoast in northern Taiwan. Open Forum Infect Dis. 2019;6:ofz447. PubMed https://doi.org/10.1093/ofid/ofz447

3. Lee HY, Huang CW, Chen CL, Wang YH, Chang CJ, Chiu CH. Emergence in Taiwan of novel imipenem-resistant *Acinetobacter baumannii* ST455 causing bloodstream infection in critical patients. J Microbiol Immunol Infect. 2015;48:588–96. PubMed https://doi.org/10.1016/j.jmii.2015.04.001

Appendix Table 1. Accession numbers of the genomic sequences sequenced and analyzed in this study

Strain/Isolate	Collection Date	Country/ Region	Source	Accession	Note
GB10	2019/7/3	Taiwan	food/environment	WHYQ00000000	sequenced in this study
GC64–2	2019/7/3	Taiwan	food/environment	WHYP00000000	sequenced in this study
GC66–1	2019/7/3	Taiwan	food/environment	WHYO00000000	sequenced in this study
GC67–2	2019/7/3	Taiwan	food/environment	WHYN00000000	sequenced in this study
GC68–1	2019/7/3	Taiwan	food/environment	WHYM00000000	sequenced in this study
M-2589	2018/8/21	Taiwan	food/environment	WHYL00000000	sequenced in this study
M-2592	2018/8/21	Taiwan	food/environment	WHYK00000000	sequenced in this study
M-3471	2018/3/31	Taiwan	food/environment	CP045458-CP045460	sequenced in this study
M-3851	2017/6/15	Taiwan	food/environment	CP045461-CP045463	sequenced in this study
M-3853	2017/6/15	Taiwan	food/environment	WHYJ00000000	sequenced in this study
M-4763	2019/7/3	Taiwan	food/environment	WHYI00000000	sequenced in this study
M-4847	2018/10/3	Taiwan	food/environment	WHYH00000000	sequenced in this study
M-4949	2018/1/11	Taiwan	food/environment	WHYG00000000	sequenced in this study
M-5351	2017/8/3	Taiwan	food/environment	WHYF00000000	sequenced in this study
M-5360	2017/8/3	Taiwan	food/environment	CP045509-CP045512	sequenced in this study
M-5365	2017/8/3	Taiwan	food/environment	WHYE00000000	sequenced in this study
M-6525	2019/7/25	Taiwan	food/environment	WHYD00000000	sequenced in this study
M-6697	2018/4/16	Taiwan	food/environment	WHYC00000000	sequenced in this study
M-6699	2018/4/16	Taiwan	food/environment	WHYB00000000	sequenced in this study
M-7537	2018/8/2	Taiwan	food/environment	WHYA00000000	sequenced in this study
M-9196	2017/8/18	Taiwan	food/environment	WHXZ00000000	sequenced in this study
M-9750	2019/6/13	Taiwan	food/environment	WHXY00000000	sequenced in this study
Sal-1135	2012/6/22	Taiwan	human	CP045464	sequenced in this study
Sal-2097	2013/11/1	Taiwan	human	CP045465	sequenced in this study
Sal-2955	2015/6/13	Taiwan	human	WHXX00000000	sequenced in this study
Sal-3348	2015/12/5	Taiwan	human	WHXW00000000	sequenced in this study
Sal-3381	2015/12/19	Taiwan	human	WHXV00000000	sequenced in this study
Sal-3386	2015/12/21	Taiwan	human	WHXU00000000	sequenced in this study
Sal-3389	2015/12/21	Taiwan	human	WHXT00000000	sequenced in this study
Sal-3343	2015/12/3	Taiwan	human	WHXS00000000	sequenced in this study
Sal-3660	2016/6/29	Taiwan	human	WHXR00000000	sequenced in this study
Sal-3805	2016/7/23	Taiwan	human	WHXQ00000000	sequenced in this study
Sal-3824	2016/7/28	Taiwan	human	WHXP00000000	sequenced in this study
Sal-3892	2016/8/17	Taiwan	human	WHXO00000000	sequenced in this study
Sal-3897	2016/8/20	Taiwan	human	WHXN00000000	sequenced in this study
Sal-3930	2016/9/1	Taiwan	human	WHXM00000000	sequenced in this study
Sal-3944	2016/9/5	Taiwan	human	WHXL00000000	sequenced in this study
Sal-3948	2016/9/6	Taiwan	human	CP045513-CP045514	sequenced in this study
Sal-3973	2016/9/15	Taiwan	human	CP045466-CP045467	sequenced in this study
Sal-3985	2016/9/10	Taiwan	human	WHXK00000000	sequenced in this study
Sal-3991	2016/9/19	Taiwan	human	WHXJ00000000	sequenced in this study
Sal-3993	2016/9/11	Taiwan	human	WHXI00000000	sequenced in this study
Sal-3997	2016/9/13	Taiwan	human	WHXH00000000	sequenced in this study
Sal-4162	2016/10/25	Taiwan	human	WHXG00000000	sequenced in this study
Sal-4179	2016/10/20	Taiwan	human	WHXF00000000	sequenced in this study

Strain/Isolate	Collection Date	Country/ Region	Source	Accession	Note
Sal-4221	2016/11/23	Taiwan	human	WHXE00000000	sequenced in this study
Sal-4295	2017/1/11	Taiwan	human	CP045515	sequenced in this study
Sal-4377	2017/3/26	Taiwan	human	WHXD00000000	sequenced in this study
Sal-4420	2017/5/5	Taiwan	human	WHXC00000000	sequenced in this study
Sal-4478	2017/5/22	Taiwan	human	WHXB00000000	sequenced in this study
Sal-4494	2017/5/31	Taiwan	human	WHXA00000000	sequenced in this study
Sal-4499	2017/6/2	Taiwan	human	WHWZ00000000	sequenced in this study
Sal-4518	2017/6/4	Taiwan	human	WHWY00000000	sequenced in this study
Sal-4550	2017/6/16	Taiwan	human	WHWX00000000	sequenced in this study
Sal-4567	2017/6/21	Taiwan	human	WHWW00000000	sequenced in this study
Sal-4583	2017/6/28	Taiwan	human	WHWV00000000	sequenced in this study
Sal-4627	2017/7/14	Taiwan	human	WHWU00000000	sequenced in this study
Sal-4698	2017/8/3	Taiwan	human	WHWT00000000	sequenced in this study
Sal-4737	2017/8/17	Taiwan	human	CP045516-CP045517	sequenced in this study
Sal-4762	2017/8/21	Taiwan	human	WHWS00000000	sequenced in this study
Sal-4873	2017/9/15	Taiwan	human	WHWR00000000	sequenced in this study
Sal-4995	2017/10/16	Taiwan	human	WHWQ00000000	sequenced in this study
Sal-5067	2017/11/16	Taiwan	human	WHWP00000000	sequenced in this study
Sal-5091	2017/11/24	Taiwan	human	CP045518-CP045521	sequenced in this study
Sal-5131	2017/12/6	Taiwan	human	WHWO00000000	sequenced in this study
Sal-5147	2017/12/14	Taiwan	human	WHWN00000000	sequenced in this study
Sal-5186	2018/1/10	Taiwan	human	WHWM00000000	sequenced in this study
Sal-5191	2018/1/10	Taiwan	human	WHWL00000000	sequenced in this study
Sal-5196	2018/1/19	Taiwan	human	WHWK00000000	sequenced in this study
Sal-5200	2018/1/22	Taiwan	human	WHWJ00000000	sequenced in this study
Sal-5217	2018/2/22	Taiwan	human	WHWI00000000	sequenced in this study
Sal-5226	2018/2/27	Taiwan	human	WHWH00000000	sequenced in this study
Sal-5240	2018/3/16	Taiwan	human	WHWG00000000	sequenced in this study
Sal-5242	2018/3/19	Taiwan	human	WHWF00000000	sequenced in this study
Sal-5328	2018/5/21	Taiwan	human	WHWE00000000	sequenced in this study
Sal-5379	2018/6/7	Taiwan	human	WHWD00000000	sequenced in this study
795421	Aug. 2019	United Kingdom	human	AAKCLH01	downloaded from public database
300680	Sep. 2016	United Kingdom	human	AAHNES01	downloaded from public database
PNUSAS010879	Missing	USA	human	AAEHEA01	downloaded from public database
PNUSAS011492	Missing	USA	human	AAGSRM01	downloaded from public database
PNUSAS038936	Missing	USA	human	AAIICK01	downloaded from public database
PNUSAS051059	Missing	USA	human	AADNYC01	downloaded from public database
PNUSAS051057	Missing	USA	human	AADTUF01	downloaded from public database
PNUSAS068759	Missing	USA	human	AADAOT01	downloaded from public database
FDA00008841	2015/2/20	Philippines	food/environment	AAGLTB01	downloaded from public database
227024	Feb. 2016	United Kingdom	human	AAHNEA01	downloaded from public database

Appendix Table 2. *Salmonella* serovars isolated from 438 food samples obtained from traditional markets and supermarkets in northern Taiwan in 2017–2019.

Food	Positive Rate	Serotype (n)
Raw Pork	50.7% (75/148)	S. Agona (10); *S.* Anatum (11); *S.* Corvallis (1); *S.* Derby (16); *S.* Give (5); *S.* Goldcoast (1); *S.* Kentucky (3); *S.* Livingstone (3); *S.* London (8); *S.* Mbandaka (2); *S.* Muenster (4); *S.* Newport (2); *S.* Potsdam (1); *S.* Rissen (1); *S.* Typhimurium (3); *S.* Weltevreden (4)
Raw Chicken	36.6% (34/93)	*S.* Albany (6); *S.* Anatum (4); *S.* Brancaster (3); *S.* Derby (1); *S.* Enteritidis (4); *S.* Goldcoast (1); *S.* Kentucky (6); *S.* Livingstone (2); *S.* Muenster (2); *S.* Schwarzengrund (2); *S.* Thompson (1); *S.* Typhimurium (2)
Raw Beef	3.7% (1/27)	*S.* Anatum (1)
Raw Duck	100% (1/1)	*S.* Albany (1)
Egg	0% (0/46)	ND
Vegetable	5.2% (5/96)	*S.* Albany (1); *S.* Derby (2); *S.* Kaitaan (1); *S.* Zigong (1)
Seafood	6.7% (1/15)	*S.* Albany (1)
Fruit	0% (0/12)	ND

ND, not detected.

Appendix Table 3. *Salmonella* isolation rate between supermarkets and traditional markets in northern Taiwan in 2017–2019*

Sample	Source (n)	Positive (n)	Negative (n)	Positive Rate (%)	*P*-value
Pork	Supermarket (36)	4	32	11.1	<0.001
	Traditional Market (112)	71	41	63.4	
Chicken	Supermarket (36)	7	29	19.4	0.006
	Traditional Market (57)	27	30	47.4	
Duck	Supermarket (0)	ND	ND	ND	NA
	Traditional Market (1)	1	0	100	
Beef	Supermarket (12)	0	12	0	NA
	Traditional Market (15)	1	14	0	
Seafood	Supermarket (8)	0	8	0	0.268
	Traditional Market (7)	1	6	14.3	
Egg	Supermarket (11)	0	11	0	NA
	Traditional Market (35)	0	35	0	
Vegetable	Supermarket (37)	0	37	0	0.069
	Traditional Market (59)	5	54	8.5	
Fruit	Supermarket (4)	0	4	0	NA
	Traditional Market (8)	0	8	0	

*ND, not detected; NA, not applicable.

Appendix Table 4. Case-control survey for *Salmonella*-infected infants under 1 y of age in Chang Gung Memorial Hospital, Taiwan in 2019.

Items	*Salmonella* (n = 20)		Healthy (n = 80)						
	n	%	n	%	Odds Ratio	95% Low	95% High	p-value	FDR*
Contact									
Share toys with other people	4	20%	13	16%	1.288	0.371	4.481	0.69	0.794
Share pillow, comforter or mattress with other people	10	50%	35	44%	1.286	0.482	3.431	0.615	0.755
Surroundings									
Purchase bulk eggs without washing	11	55%	19	24%	3.924	1.414	10.886	0.006	0.081
Traditional market as the major pork shopping place	11	55%	20	25%	3.667	1.328	10.127	0.009	0.081
Home cooking/Vegetable	18	90%	59	74%	3.203	0.684	14.994	0.122	0.302
Home cooking/Rice, noodle, other grains	18	90%	61	76%	2.803	0.596	13.194	0.177	0.376
Home cooking/Pork	14	70%	38	48%	2.579	0.900	7.386	0.072	0.249
Cook raw food and delicatessen with the same chop board	10	50%	23	29%	2.478	0.910	6.746	0.071	0.249
Main caregiver are grandparents or other elders	8	40%	17	21%	2.471	0.871	7.009	0.083	0.249
Traditional market as the major egg shopping place	8	40%	20	25%	2.000	0.716	5.590	0.181	0.376
Home cooking/Eggs	13	65%	46	58%	1.373	0.495	3.807	0.542	0.752
Diet									
Rinse or wash raw meat before cooking	18	90%	50	63%	5.400	1.170	24.923	0.018	0.122
Process raw meat before feeding infants	10	50%	15	19%	4.333	1.530	12.271	0.004	0.081
Eat banana	5	25%	8	10%	3.000	0.861	10.452	0.074	0.249
Eat pork	6	30%	15	19%	1.857	0.613	5.629	0.269	0.484
Eat apple	7	35%	18	23%	1.855	0.644	5.343	0.248	0.478
Use milk powder, rice cereal or malt extract in 30 d	5	25%	14	18%	1.571	0.490	5.037	0.444	0.749
Wash and air-dry scoop after feeding milk powder, rice cereal or malt extract	11	55%	37	46%	1.420	0.531	3.802	0.484	0.752
Eat rice cereal	4	20%	12	15%	1.417	0.404	4.973	0.585	0.752
Infants need to be fed by caregivers	15	75%	55	69%	1.364	0.446	4.167	0.585	0.752
Eat shrimp	7	35%	23	29%	1.334	0.472	3.770	0.585	0.752
Drinking water after water filter and boiling	5	25%	17	21%	1.235	0.393	3.882	0.717	0.794
Frequency of handwashing before feeding infants (>75%)	6	30%	21	26%	1.204	0.410	3.540	0.735	0.794
Diet/Dairy Products									
Milk powder	17	85%	66	83%	1.202	0.310	4.665	0.790	0.820
Breast milk	3	15%	26	33%	0.367	0.099	1.363	0.123	0.302
Purchase Source									
Traditional markets	14	70%	34	43%	3.157	1.100	9.058	0.028	0.151
Supermarkets	8	40%	34	43%	0.902	0.332	2.448	0.839	0.839

*FDR, False Discovery Rate.

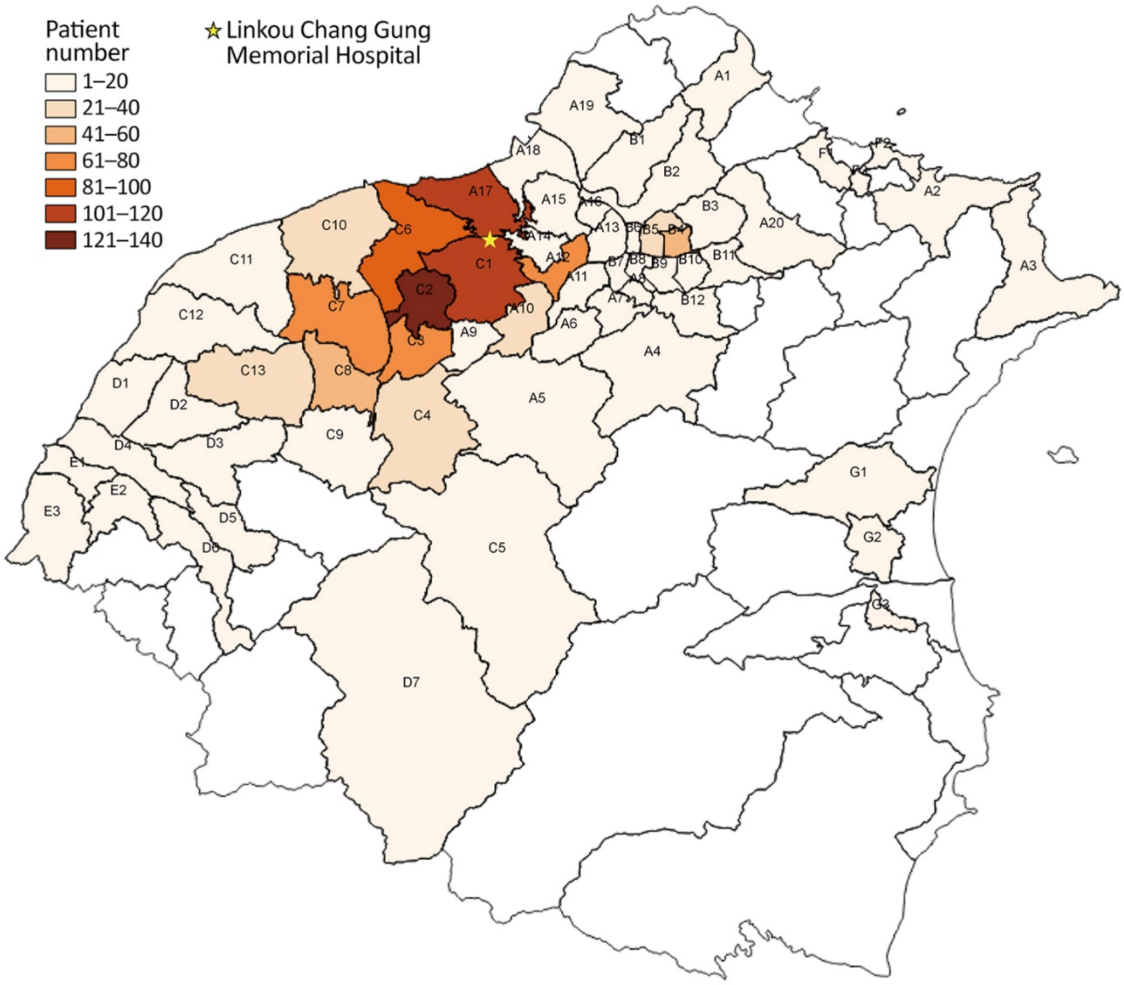

Appendix Figure 1. Food samples surveyed for *Salmonella* from supermarkets and traditional markets of eight districts with higher density of *Salmonella* infection in New Taipei City and Taoyuan City of northern Taiwan. *S.* Anatum were isolated from traditional markets and supermarkets of Linkou District (A17) and Xinzhuang District (A12), and from traditional markets of Taoyuan District (C2), Zhongli District (C7), and Guishan District (C1).

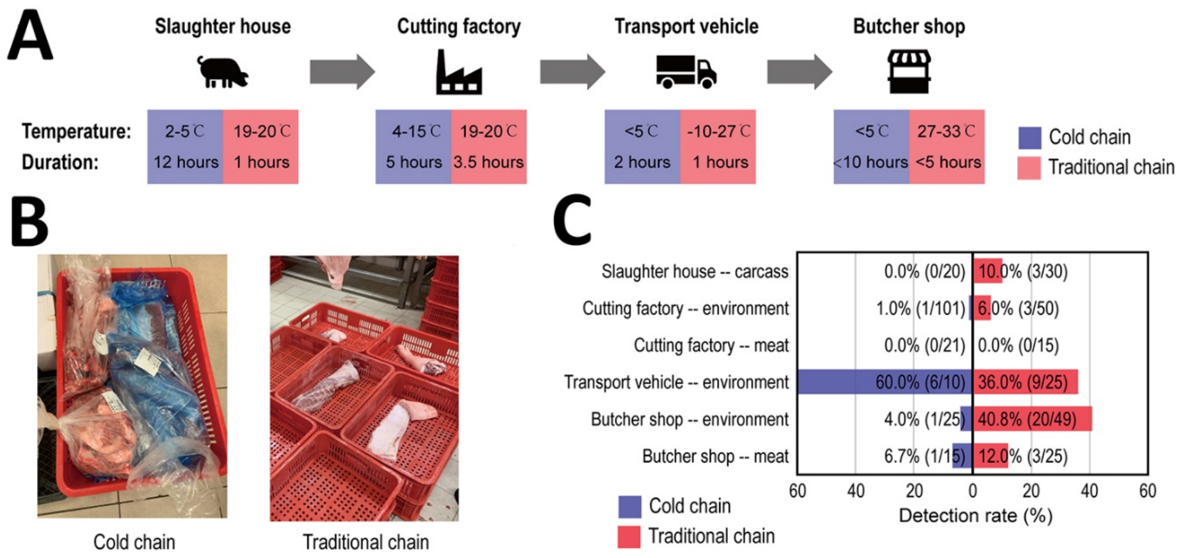

Appendix Figure 2. A comparison of *Salmonella* detection rates between the cold chain and traditional chain. A) Differences of temperature and transportation time between the two transportation chains. B) Different packaging manners employed during transportation. C) A comparison of detection rates of *Salmonella* spp. between the cold and traditional transportation chains.
